# Editorial: New Insights Into Renal Fibrosis and Therapeutic Effects of Natural Products

**DOI:** 10.3389/fphar.2022.923773

**Published:** 2022-05-16

**Authors:** Ying Xu, Danqian Chen, Zhiyong Guo

**Affiliations:** ^1^ Department of Nephrology, Changhai Hospital, Naval Medical University, Shanghai, China; ^2^ Faculty of Life Science & Medicine, Northwest University, Xi’an, China

**Keywords:** editorial, renal fibrosis (RF), natural producct, therapeutic effect, new insight

Renal fibrosis is the progressive and complicated process manifested by histological aberrance and functional decline in the kidney. The pathogenesis involves multiple molecular pathways and cellular targets leading to myofibroblast activation and accumulation of extracellular matrix, which is in response to excessive epithelial injury and inflammation. Formation and exacerbation of fibrosis during the development of chronic kidney disease (CKD) is the common pathway to end-stage renal failure. However, few interventions are available that specifically target the pathogenesis of renal fibrosis. Emerging evidences prove that natural product therapy directly targets the pathogenesis of renal fibrosis, and exhibits beneficial effects in clinical. Further research (such as multi-omics studies and network pharmacology) is urgently needed to investigate the puzzle from the active compounds to underlying mechanisms and therapeutic targets of natural product against renal fibrosis.

The Research Topic intends to highlight the latest advances from the active compounds to underlying mechanisms and therapeutic targets molecular mechanism of natural product against renal fibrosis. The issue includes 25 articles that is contributed by more than 200 authors in the fields of renal pharmacology. We have generated a collaborative discussion that facilitated the development of new mechanisms, new therapeutic targets, and candidate drugs from natural product against renal fibrosis.

New mechanisms of renal fibrosis and the protective effects of natural product have been investigated and uncovered. Fibrosis-related signaling pathways are the common therapeutic targets, especially TGF-β pathway. Zheng et al. illustrate that astragalus polysaccharide extract plays a beneficial role in reducing renal inflammation and fibrosis and in improving renal function by regulating the TGF-β/ILK pathway in hypertensive mice. Ren et al. confirm that natural flavonoid pectolinarigenin alleviates renal fibrosis to delay hyperuricemic nephropathy via suppressing TGFβ/SMAD3 and JAK2/STAT3 signaling pathways. By targeting non-TGF-β pathway, Yu et al. report the medical leech saliva extract, hirudin, protects the kidney from fibrotic injury by ameliorating renal autophagy impairment via PI3K/Akt pathway. Liu et al. demonstrate that quercetin alleviates podocytes apoptosis *in vitro* and *in vivo* by regulating the EGFR pathway, providing a novel approach to reveal the therapeutic mechanisms of quercetin against DN. Gong et al. identify proanthocyanidins (OPC) as active compounds of grape seeds. OPC exhibits beneficial protection against cadmium-induced DN in multidimensional aspects by the regulation of oxidative-antioxidative status, metal-binding ability, mediation of the levels of essential elements, and activation of the p38 MAPK and Keap1/Nrf2 signaling pathways.

Additionally, metabolic regulation and kidney-gut axis function as a promising therapeutic target against renal fibrosis. Ma et al. prove that farrerol reverses oxidative stress, inflammation, and fibrosis in renal tubular epithelial cells by activating Nrf2 and subsequently increasing PINK/Parkin-mediated mitophagy and eliminating damaged mitochondria. Xiang et al. show that the restoration of PPARα activity delays diabetic nephropathy progression and attenuates lipid metabolism disorders by downregulating miR-21 expression to improve mitochondrial function. Li et al. report that maintaining the balance of mitochondrial dynamics exhibits renoprotection in kidney by inhibiting mitochondrial fission and promoting mitochondrial fusion via the downregulation of the primary mediator proteins of mitochondrial fission (Drp1 and Fis1) and the upregulation of fusion proteins (Opa1 and Mfn1). Zhang et al. carries out the serum metabonomics analysis of patients with diabetic kidney disease and shows the potential role of specific gut microbiota in the progression of renal fibrosis and diabetic kidney disease that involves the dysfunction of phenylalanine and tryptophan metabolisms. This study suggests the kidney-gut axis functions as a potential therapeutic target of renal fibrosis.

Since Chinese herbal medicine exhibits beneficial effects against renal fibrosis, the lack of randomized controlled trial and clear mechanism hinder natural product to pass modern assessments. Here, Shao et al. summarized five Chinese herbal medicines with sufficient clinical efficacy, high frequency of use, and well-studied mechanism, including *Abelmoschus manihot* and Huangkui capsule, *Salvia miltiorrhiza* and its components (tanshinone II A, salvianolic acid A and B); *Rhizoma coptidis* and its monomer berberine; *Tripterygium wilfordii* and its components (triptolide, tripterygium glycosides); *Kudzu* root *Pueraria* and its monomer Puerarin. The researches of these five Chinese herbal medicines set the study pattern for natural product against renal fibrosis, which are the promising candidate for widespread and standardized application.

Beyond to five Chinese herbal medicines, studies from the present issue provide the clinical evidences of natural products against renal fibrosis and explore underlying mechanisms. Mahuang Fuzi and Shenzhuo Decoction (MFSD), a Chinese herbal formula, is a promising candidate for renal fibrosis and CKD treatment in clinial. Dong et al. designed a multicenter, nonrandomized, single-arm clinical trial to explore the clinical effects of MFSD on idiopathic membranous nephropathy (MN), presenting an inspiring result that MFSD has significantly beneficial effects on idiopathic MN treatment, and has the same beneficial effects on patients with MN who are newly treated and who accepted with immunosuppressive therapy without remission. Further study from the same group by Gao et al. investigates the main active compounds of MFSD by high-performance liquid chromatography-mass spectrometry (HPLC-MS) and more than 30 active compounds are identified. These active compounds alleviate podocyte injury by modulating autophagy-related protein and Wnt/β-catenin pathway, indicating autophagy and Wnt/β-catenin pathway as potential targets of MFSD for MN treatment. Zhang et al. demonstrate the anti-fibrotic and anti-inflammatory effects of Bupi Yishen Formula *in vivo* and *in vitro* by suppressing TLR4-mediated NF-κB signaling.The study of Luo et al. reveals the anti-oxidant and anti-inflammatory ability of Shenkang injection (SKI) in kidney, and identifies chrysophanol, emodin, and rhein as active compounds against renal fibrosis via simultaneously targeting IκB/NF-κB and Keap1/Nrf2 signaling pathways. Jia et al. report TLYS decoction improves mitochondrial dynamics to attenuates renal fibrosis and renal function decline by suppressing oxidative stress and mitophagy. The comparative network pharmacology analysis is the alternative approach to explore the mechanisms of natural products with limited experiments. Chan et al. performed comparative network pharmacology to figure out the mechanism of Liu-wei-di-huang-wan. Liu-wei-di-huang-wan may ameliorate fibrosis, angiogenesis, inflammation, disease susceptibility, and oxidative stress via modulating TNF signaling pathway, which could be validated through clinical trials.

In conclusion, the collection of 25 articles in the Research Topic contributes to better understanding of natural products against renal fibrosis from the active compounds to underlying mechanisms and therapeutic targets. These studies provide promising and potential candidates against renal fibrosis by modulating novel pathways in clinical trial and animal experiment. Accompanying with the evolution of high throughput screening method and model, novel candidates and therapeutic targets will emerge to facilitate the drug discovery, which hold great potential for treatment with renal fibrosis.

